# Monarch butterflies (*Danaus plexippus*) only use magnetic cues for migratory directionality with orientation re-calibrated by coldness

**DOI:** 10.1371/journal.pone.0328737

**Published:** 2025-08-13

**Authors:** Samuel A. Shively-Moore, Stephen F. Matter, Patrick A. Guerra

**Affiliations:** 1 Department of Biological Sciences, University of Cincinnati, Cincinnati, Ohio, United States of America; 2 School of Environment and Sustainability, University of Cincinnati, Cincinnati, Ohio, United States of America; Chulalongkorn University, THAILAND

## Abstract

Each fall, Eastern North American monarch butterflies (*Danaus plexippus*) leave their northern range and migrate to their overwintering sites high atop mountains in central Mexico. Although monarchs primarily rely on the use of a bidirectional time-compensated sun compass to maintain southwards directionality *en route* to Mexico, on overcast sky days when directional daylight cues are unavailable, monarchs can use an inclination-based magnetic compass to maintain correct directionality. As compass cues can only be used to determine direction, monarchs must use other mechanisms for recognizing, locating, and ultimately stopping at their overwintering sites. Although previous work found no evidence of monarchs using a fine-scale magnetic map for locating their specific overwintering sites, monarchs might still use magnetic cues in a general sense, such as when recognizing that they have overshot their destination or have gone off course. Here, using righting response orientation trials, we show that fall monarchs maintain equatorward (southward) orientation even when tested under artificially generated magnetic field conditions consistent with either their overwintering sites or magnetic conditions geographically south of these sites. We also found that fall migrants exposed to overwintering-like coldness reverse their orientation poleward (northward). This result indicates that the monarch’s magnetic compass is also recalibrated by the cold temperature microenvironment at the overwintering sites, as has been shown previously with its time-compensated sun compass. Our results indicate that migratory monarchs must use other cues for locating and stopping at their migratory destination. Our discovery that coldness recalibrates multiple compass mechanisms in a long-distance migratory species underscores the threat of climate change and corresponding increasing temperatures on animal migration.

## Introduction

Many animal species perform long-distance migrations using environmental sensory cues that provide them with information that facilitates their arrival at their destinations [1,2]. An example of an environmental sensory cue used by animals for navigation during long-distance migration is the Earth’s magnetic field [3], with diverse taxa such as marine animals (e.g., sea turtles [4], salmonid fishes [5], and spiny lobsters [6]), birds (e.g., European robins [7] and pigeons [8]), and insects (e.g., monarch butterflies [9,10] and bogong moths [11]) employing geomagnetic cues during long-distance travel. Geomagnetic cues can produce two types of information. First, individuals can use geomagnetic cues as part of a magnetic compass, in which geomagnetic parameters provide directional information that allows individuals to maintain the correct migratory heading [4]. Second, animals can use geomagnetic cues for positional information that allows them to assess their geographic location, producing a map sense that allows them to discern their current location relative to their destination [3,4].

Each fall, Eastern North American monarch butterflies (*Danaus plexippus*) leave their summer breeding grounds in southern Canada and the northern tier of the United States and migrate southwards, flying to specific overwintering sites in central Mexico that are found at high elevation [12]. During the spring, these same individuals become spring remigrants. The remigrants leave Mexico and fly north to start repopulating the northern areas of their range [12,13]. During both legs of their journey, migratory monarchs primarily rely on a bidirectional time-compensated sun compass to guide flight in the correct migratory direction [14–16]. In the fall, the time-compensated sun compass is tuned to maintain southward migratory flight [14]. The time-compensated sun compass is then recalibrated in monarchs from southwards to northwards flight in the spring via exposure to cold conditions during the overwintering period at the overwintering sites. Without exposure to coldness, migratory monarchs maintain southward oriented flight [17].

On days when directional daylight cues are unavailable, e.g., overcast conditions, fall migratory monarchs can use a backup light-sensitive inclination-based magnetic compass to maintain correct equatorward (southward) flight orientation [18]. Fall migratory monarchs were shown to possess a magnetic sense [18–20] in which migratory butterflies can detect and use geomagnetic information provided by the inclination angle of the Earth’s magnetic field to maintain correct equatorward fall migratory directionality [18,19]. Although not yet directly tested, it is likely that spring remigrants also use a recalibrated magnetic compass (tuned now for poleward flight) as a backup on overcast days during northward spring remigration. How the directionality of the magnetic compass is recalibrated is unknown.

Both the time-compensated sun compass and the magnetic compass only provide migratory monarchs with information on migratory directionality [18]. Moreover, these compass mechanisms do not provide information that allows fall migratory monarchs to recognize, locate, or stop at their overwintering sites [10]. One of the great unsolved mysteries of the iconic annual monarch migration is how first-time, naïve fall migrants find their overwintering sites. One potential navigational mechanism is that fall monarchs might use an inherited map sense based on geomagnetic cues for finding their overwintering sites each year [10]. Geomagnetic map senses can vary in their features and functions across migratory species [4]. Previous work was consistent with fall migratory monarchs not possessing a *specific fine-scale magnetic map sense* [10], in which monarchs respond to specific geomagnetic coordinates correlated with the geographic location of the overwintering sites. It remains possible, however, that fall migrants might still possess an inherited *general magnetic map sense* that assists with finding their overwintering sites [10]. Such a general map sense could allow individuals to broadly recognize their current location relative to the overwintering sites, as seen in other migratory species such as sea turtles [21]. Here, a general map sense could provide monarchs with positional information, such as if they have passed or gone off-course relative to their goal destination, thereby allowing them to course correct as necessary to approach the overwintering sites. Monarchs would then presumably use other finer-scale cues for finding their overwintering sites [2].

In this work, we tested the hypothesis that fall migratory monarchs possess an inherited general geomagnetic map sense by examining their behavior in righting response orientation trials when exposed to different artificially generated magnetic fields (artificial magnetic displacement trials [4]) as the only source of directional or positional information [19]. Similar to migratory birds, fall migratory monarchs possess a migratory syndrome, with one aspect of the syndrome the propensity of migrants to display a preference to move and orient in the correct migratory direction even when not actively migrating [19]. For example, migratory birds will display Zugunruhe, manifested as migratory restlessness in which birds will move and orient in the appropriate seasonal migratory direction when held in captivity (e.g., when tested in Emlen funnels [22]), using the magnetic field for directionality [23]. Fall migratory monarchs display behavior similar to Zugunruhe in birds during righting response trials, in which monarchs display a preference for being upright and will immediately right themselves to a vertical head up position [19]. Here, after righting themselves, monarchs will then shift and orient in the appropriate fall migratory direction (i.e., equatorward), using specific magnetic field cues (inclination angle) for directionality [19].

**Fig 1:**
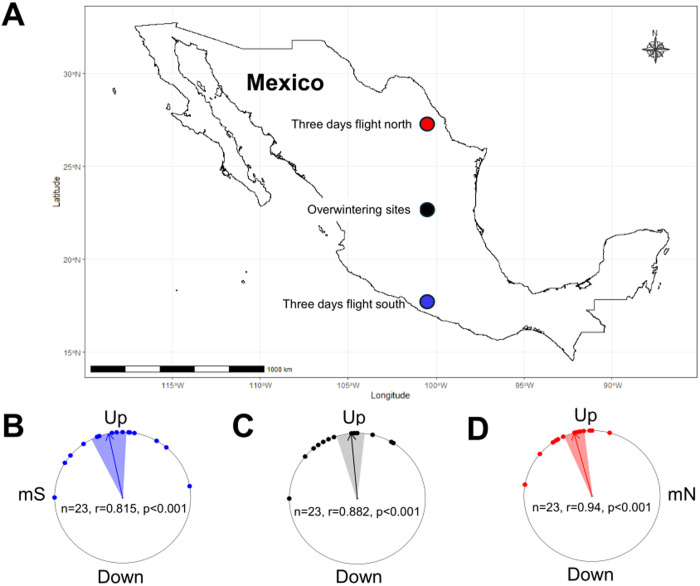
The effect of magnetic field parameters on the direction of orientation of fall migratory monarchs. (A) In artificial displacement trials, monarchs were tested in three distinct artificial magnetic field conditions that corresponded to different geographical locations in Mexico: (1) three days flight south of the overwintering sites (Acapulco, Guerrero, Mexico; blue circle), (2) the overwintering sites (center of the Monarch Butterfly Biosphere Reserve, Michoacán-Mexico State, Mexico; black circle), and (3) three days flight north of the overwintering sites (San Luis Potosi, San Luis Potosi, Mexico; red circle). (B-D) Righting response orientation bearings of individual 2023 fall migratory monarchs (red, black, and blue dots on circle diagram, n = 23 for all) tested in all three trial conditions. Across all treatment conditions, monarchs maintained mean headings with equatorward directionality. Statistics from the circle diagrams show the number of individuals tested (n), the length of the mean vector (r), and the Rayleigh’s test significance (p). For each circle diagram, each dot indicates the heading of an individual butterfly, the arrow indicates the mean group orientation, shaded area is the confidence interval, the color corresponds to the location that shares the same colored circle in (A), mN is magnetic north, and mS is magnetic south. The map in Fig 1A was made using the R package ggplot2 (https://ggplot2.tidyverse.org).

We began our study by testing fall migratory monarchs captured on-the-wing in the Greater Cincinnati Area in righting response trials, during which monarchs were exposed to artificial magnetic fields that mimic geographic conditions south of their overwintering sites in Mexico (Fig 1). In these artificial displacement trials [19], fall migrants tested under these conditions would be naïve to these magnetic cues, eliminating the effects of any direct experience or learning on their behavior. Artificial displacement trials are considered a litmus test for an animal possessing a geomagnetic map sense [2,4]. We predicted that if monarchs have an inherited general magnetic map sense to navigate to their overwintering sites, monarchs will orient polewards in the perceived direction of where the overwintering sites would be, when tested under unfamiliar conditions south of the overwintering sites. This response would be similar to those observed in species such as sea turtles, spiny lobsters, salmonid fish, and birds that have a magnetic map sense and that adjust their movement in response to where they perceive to be relative to their goal destination [4]. For monarchs, these magnetic conditions would be consistent with overshooting their destination or being off course relative to their overwintering sites.

We also tested monarchs under magnetic field conditions that mimicked the geographic location of the overwintering sites themselves (Fig 1), to examine if they would alter their behavior in response to magnetic cues indicative of the overwintering sites. We predicted that if monarchs respond to magnetic cues associated with the overwintering sites as part of a magnetic map mechanism for recognizing and finding these sites (e.g., beacon cues [2]) or treat magnetic cues as navigational landmarks [24], monarchs might shift their directionality or cease orienting in an equatorwards manner in righting response trials [19], comparable to the reduced southwards directional flight behavior seen in monarchs tested in flight simulator trials in Mexico [25]. Within this context, the shift in behavior when exposed to magnetic cues consistent with those of the overwintering sites, might trigger or precede the onset of the use of other cues (visual or olfactory [26]) or mechanisms (e.g., microhabitat selection [27]) for stopping at the overwintering sites and the formation of overwintering roosts [13].

As a control, we tested monarchs under artificial magnetic field conditions consistent with a location geographically north of the overwintering sites (Fig 1). Under these conditions, we predicted that monarchs would use the magnetic field to orient equatorwards in the direction of the overwintering sites, as seen previously in fall monarchs that were tested in flight simulator [18] and righting response trials [19].

Finally, by prematurely exposing fall monarchs to overwintering-like coldness, we examined if coldness would similarly recalibrate the magnetic response in monarchs as seen with the time-compensated sun compass [17]. Here, we predicted that fall monarchs exposed to coldness would display poleward orientation when tested under magnetic field conditions north of the overwintering sites, and that they would be responding to the inclination angle of the magnetic field. In contrast, fall monarchs not exposed to coldness would maintain the expected equatorward orientation in trials. If migratory monarchs do shift their magnetosensation behavior when exposed to overwintering-like coldness, this would support the use of magnetic cues by remigrants during the return migration polewards in the spring. Monarch magnetosensation and orientation would therefore be another environmentally tuned bidirectional sensory mechanism used during migration.

## Results and discussion

### General geomagnetic map sense

We first asked if fall migratory monarchs possessed an inherited general geomagnetic map sense for assisting with navigating to their overwintering sites. Monarchs use magnetic cues as a part of their inclination-based magnetic compass to orient towards their overwintering sites [18]. A general map sense would provide monarchs with the necessary positional information that can guide them to their overwintering sites when tested under unfamiliar conditions as seen in other migratory species that use magnetic map cues [2]. Similarly, a general map sense would facilitate the ability of monarchs to course correct if they have gone the wrong direction relative to their overwintering sites, e.g., overshot their destination. To assess whether migratory monarchs possess a general map sense, fall migratory monarchs from 2023 were captured and subjected to righting response trials under three artificial magnetic field conditions: (1) three days flight south of their overwintering sites (3DS); (2) at their overwintering sites (OWS); and (3) three days flight north of their overwintering sites (3DN) (Fig 1A). To the best of our knowledge, this study is the first to specifically test monarchs using geomagnetic parameters at and south of the overwintering sites (whether trials *in situ* or via artificial displacement trials as in this study).

For monarchs, magnetic conditions south of the overwintering sites would be consistent with overshooting or going off course relative to their destination. We predicted that if monarchs possess a general map sense, they would orient polewards if they were capable of recognizing that they were south of the overwintering sites in our righting response trials. Monarchs tested under conditions south of the overwintering sites significantly oriented as a group (3DS – Rayleigh’s test: Z = 15.262, α = 348°, r = 0.815, n = 23, p < 0.001), but exhibited an equatorward orientation (3DS – V-test: u = 5.403, α = 348°, r = 0.815, n = 23, p < 0.001) (Fig 1B). These results show that monarchs use magnetic cues for compass orientation only and do not possess a general map sense that uses magnetic cues.

When tested at the overwintering sites trial condition, we anticipated that monarchs would shift or cease orientation, if they possessed a general map sense. Under this trial condition, using their general map sense monarchs might recognize that they are at the overwintering sites and adjust their flight behavior accordingly [2] or they would display altered, disoriented flight behavior as observed in previous flight studies conducted at the overwintering sites [25]. In this treatment condition, monarchs were similarly significantly oriented as a group (OWS – Rayleigh’s test: Z = 17.911, α = 355°, r = 0.882, n = 23, p < 0.001) with equatorward directionality (OWS – V-test: u = 5.961, α = 355°, r = 0.882, n = 23, p < 0.001) (Fig 1C). These results re-affirm that monarchs do not possess a fine-scale map [10] and that they only use magnetic cues for directionality, since they show no indication of recognizing they were at the overwintering sites (and no change in behavioral response) based on magnetic cues alone.

We predicted that monarchs would orient equatorwards at 3DN conditions and used this as a control condition. Consistent with previous work [19], we found that fall monarchs oriented equatorwards when presented with magnetic conditions north of their overwintering sites. In this treatment condition, monarchs significantly oriented as a group (3DN – Rayleigh’s test: Z = 20.309, α = 345°, r = 0.94, n = 23, p < 0.001) in the predicted equatorward direction (3DN – V-test: u = 6.142, α = 345°, r = 0.94, n = 23, p < 0.001) (Fig 1D).

We found that fall migrant monarchs oriented equatorwards when tested with an artificial magnetic inclination angle, as has been observed when presented with a magnetic inclination angle in other flight and righting response trials [18,19]. Moreover, regardless of which magnetic conditions the monarchs were tested at (3DS, OWS, or 3DN), the mean orientation of monarchs was not significantly different across each condition and monarchs were all oriented equatorwards regardless of their proximity and relationship with the overwintering sites (Hotelling’s Paired Tests – 3DS & OWS: F = 0.598, n = 23, p = 0.559; 3DS & 3DN: F = 1.986, n = 23, p = 0.162; OWS & 3DN: F = 2.177, n = 23, p = 0.138) (Figs 1B–D). Together with previous work [18,19], our results therefore show that monarchs do not use magnetic cues as part of a map sense, whether on a fine scale [10] or on a broader general scale (this study). Thus, monarchs do not display an ability to use magnetic cues for recognizing or locating their overwintering sites in central Mexico, contrary to the use of magnetic cues as part of a navigational map sense for migration that is observed in sea turtles, spiny lobsters, salmon, and birds [21]. These results confirm that monarchs use magnetic cues for directionality only and that the magnetic compass serves as a secondary mechanism to the time-compensated sun compass as it provides no specific ability to home in on the overwintering sites [28].

Compass mechanisms provide only directionality, therefore monarchs likely use other environmental cues to trigger the end of their migration or for recognizing and locating their overwintering sites, such as olfactory or visual cues emanating from these areas that facilitate site recognition [9,29], or temperature cues indicative of the microenvironment of the overwintering sites that migratory monarchs might be searching for (habitat selection [27]). One of the main threats to this migratory behavior is the deforestation and widespread reduction of overwintering habitat, which could reduce the potential signal provided by the forests letting monarchs know when to stop migrating [30].

### Coldness re-calibrates orientation

**Fig 2:**
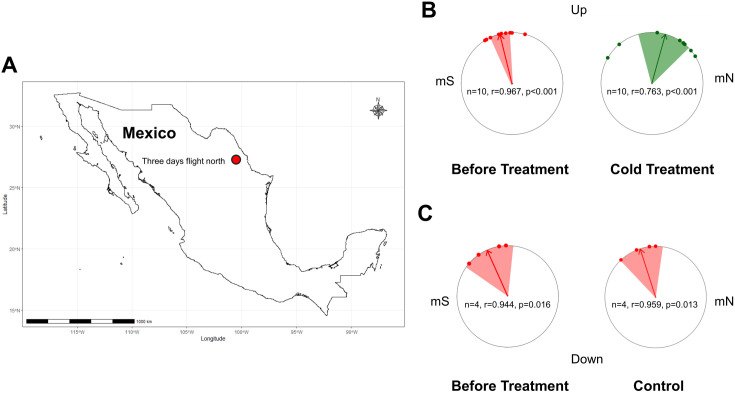
Coldness recalibrates magnetic compass orientation in migratory monarch butterflies. (A) After a 24-day period, post-cold and fall-like (control) treatment monarchs were tested at the 3DN magnetic parameters. (B) When first tested, migratory monarchs displayed a mean equatorwards heading at the 3DN magnetic field condition (left circle diagram), but post-cold treatment, the mean heading of these same monarchs was significantly shifted polewards when tested again at the 3DN condition (right circle diagram). (C) In contrast, migratory monarchs not subjected to coldness but maintained under fall-like conditions, displayed equatorward orientation in their first righting response trial at the 3DN magnetic field condition (left circle diagram) and maintained an equivalent equatorward orientation when tested a second time (right circle diagram). Statistics within the circle diagrams show the number of individuals tested (n), the length of the mean vector (r), and the Rayleigh’s test significance (p). For each circle diagram, each dot indicates the heading of an individual butterfly, the arrow indicates the mean group orientation, the shaded area is the confidence interval, mN is magnetic north, and mS is magnetic south. The map in Figure 2A was made using the R package ggplot2 (https://ggplot2.tidyverse.org).

Having established that monarchs do not use magnetic cues as part of a general map sense and confirmed that monarchs use magnetic information only for directionality as part of a magnetic compass, we then determined if monarch orientation can be recalibrated after experiencing overwintering coldness as seen with the bidirectional time-compensated sun compass [17]. North-South bidirectional orientation has been observed in multiple species of butterflies showing synchronization with the seasonal microclimate conditions [31,32]. Prior to trials, a subset of the same fall migratory monarchs we previously tested (experiments described above) were treated to cold conditions to mimic the overwintering conditions in Mexico for a 24-day period (11C:4C light:dark, L:D), and another subset of monarchs from the same group was maintained under fall-like temperature conditions for the same duration. We then examined their orientation at the 3DN magnetic conditions in a subsequent trial (Fig 2A). Recalibration of the magnetic compass was expected after exposure to these conditions, because fall migrants prematurely exposed to overwintering coldness had a recalibrated time-compensated sun compass from southward to northward orientation [18]. In contrast, fall migrants not exposed to coldness kept southward orientation when tested [18].

Before cold treatment, the subset of monarchs selected for this was significantly oriented as a group (Rayleigh’s test, pre-coldness treatment: Z = 9.351, α = 346°, r = 0.967, n = 10, p < 0.001) with the predicted equatorward directionality (V-test: u = 4.199, α = 346°, r = 0.967, n = 10, p < 0.001) (Fig 2B, left circle diagram). After treatment, the cold-treated monarchs significantly oriented as a group (Rayleigh’s test, post-coldness treatment: Z = 5.819, α = 15°, r = 0.763, n = 10, p = 0.001). Here, monarchs had now shifted their behavior to have the expected coldness-induced poleward directionality (V-test: u = 3.291, α = 15°, r = 0.763, n = 10, p < 0.001), when tested again to assay their orientation behavior post 24-day cold treatment (Fig 2B, right circle diagram). The orientations of these monarchs pre- and post- 24-day period of cold conditions were significantly different (Moore’s Paired Test: R’ = 1.085, n = 10, p < 0.05). These results show that a 24-day period of cold conditions recalibrates the magnetic orientation of monarchs, demonstrating that overwintering-like cold exposure triggers the magnetic orientation of fall migrants to shift polewards.

In both pre- and post-24-day fall-like control group conditions (no temperature change), fall migratory monarchs were consistently oriented directionally equatorward (Rayleigh’s test – pre-24-day: Z = 3.568, α = 336°, r = 0.944, n = 4, p = 0.016, Fig 2C, left circle diagram; post-24-day: Z = 3.68, α = 342°, r = 0.959, n = 4, p = 0.013, Fig 2C, right circle diagram), and there was no difference in the orientation of the butterflies across both conditions (Moore’s Paired Test: R’ = 0.492, n = 4, p > 0.5) (Fig 2C). Therefore, fall migratory monarchs will continue to orient equatorwards and do not reverse their directionality when they are maintained in fall-like conditions without exposure to coldness. This accords with observations by Guerra & Reppert [17], who showed that fall migratory monarchs not exposed to coldness will continue to orient southwards (equatorwards) when using a time-compensated sun compass, after being held in laboratory fall-like conditions and tested the following spring, at a time when conspecifics in the field were on their return leg of the migration and flying northwards (polewards).

In addition to coldness, changes in the photoperiod monarchs experience while overwintering in Mexico might also play a role in compass recalibration and remigration. For instance, the increasing photoperiod that overwintering monarchs in Mexico experience with the progression of winter and onset of spring, might play a role in inducing compass recalibration. At least for the time-compensated sun compass used by monarchs, coldness appears to be the main driver for compass recalibration, with photoperiod having little influence on shifting flight directionality from southwards to northwards [17]. It remains unknown, however, how the monarch magnetic compass might be tuned by photoperiodic cues and needs to be directly tested in future work.

Considering the key role of temperature for both time-compensated sun compass and magnetic compass recalibration, increasing global temperatures from climate change, particularly at the overwintering sites [12] poses a unique threat to these orientation mechanisms. Without the cold trigger experienced while overwintering, migratory monarchs would no longer receive the necessary signal that recalibrates their orientation mechanisms to migrate polewards the following spring. Monarchs may even continue to migrate south, even when they would usually be flying polewards. Currently, it remains unknown how increasing temperatures at the overwintering sites in central Mexico during the overwintering period might interfere with compass recalibration and the migration of Eastern monarchs. Future work can determine the thresholds for the coldness overwintering monarchs must experience, e.g., the minimum cold temperature and duration of exposure monarchs must be exposed to for successful remigration. Knowledge of these coldness thresholds can determine how increasing temperatures due to climate change might disrupt monarch migration.

**Fig 3:**
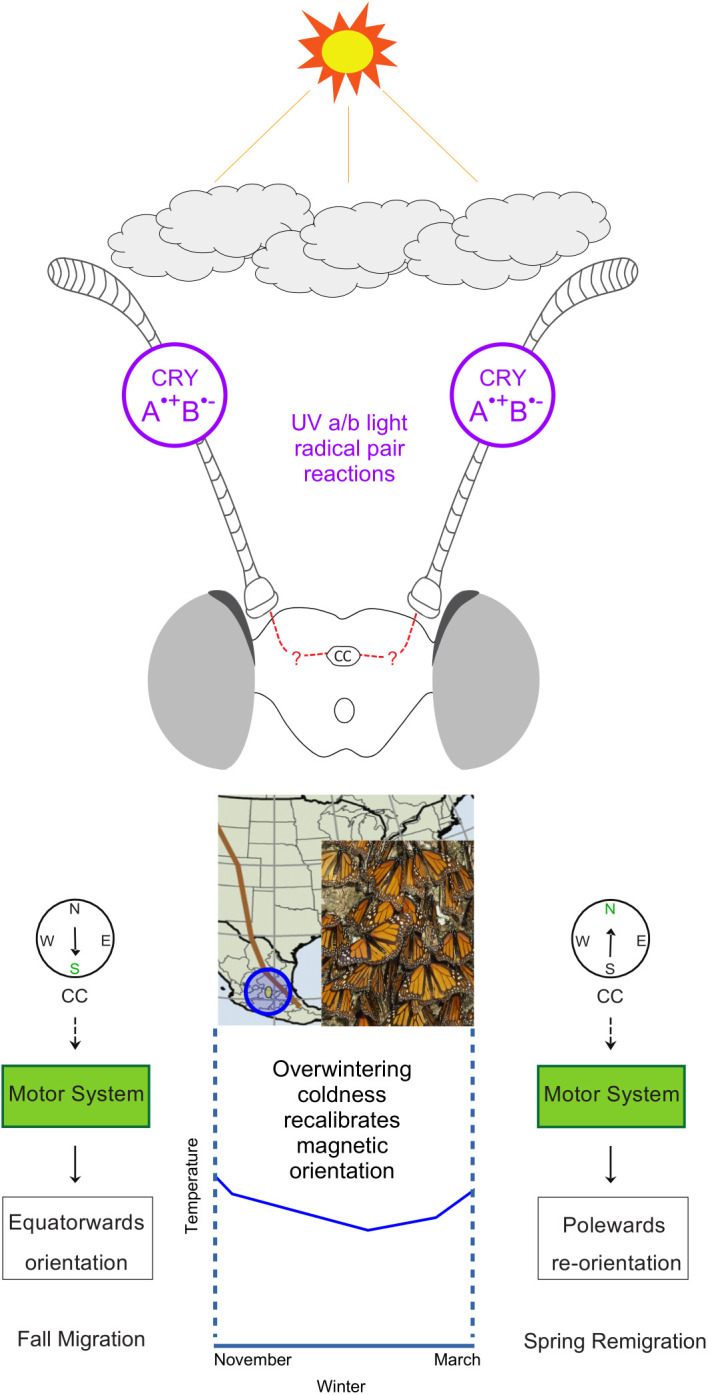
Hypothetical neural circuit of the environmentally-tuned monarch magnetic compass.

Although monarchs use environmental information to orient migratory flight, the exact mechanism for sensing temperature and responding to temperature changes related to overwintering and seasonality are less understood. Temperature in monarchs, however, has been shown to be sensed by the antenna [27], with the processing of temperature cues potentially occurring further downstream in the neural network (Fig 3) [27,33]. Ultimately, their ability to arrive at their overwintering sites is likely a combination of many cues, yet to be identified, that guide the migration behavior of the monarch butterfly [2].

A hypothetical neural circuit. The antennae have relevant magnetosensors based on cryptochrome. Information from these antennae is sent to the central complex [33]. Using magnetosensation, monarchs orient equatorwards in the fall. They overwinter (overwintering sites in yellow oval, blue area around oval denotes the potential overwintering area) from November to March in conditions cold enough to slow metabolism but continue to survive [17]. These cold conditions recalibrate their orientation so that in the spring they orient polewards [17]. As observed with the time-compensated sun compass [17], the magnetic sense of fall monarchs prematurely exposed to coldness was shifted to a polewards orientation, whereas fall monarchs not exposed to coldness maintained equatorwards directionality. Middle panel modified from Guerra and Reppert [33], containing a map of North America with the Rocky Mountains/Sierra Madre Occidental mountain ranges labelled with a brown line.

## Conclusion

In conjunction with previous work [17], our study demonstrates that at least two orientation mechanisms that facilitate monarch migration require some period of coldness exposure to recalibrate the migratory directionality of monarchs to return polewards in the spring. Normally, Eastern North American migratory monarchs orient equatorwards in the fall until they reach their overwintering sites where they experience cold conditions from November to March; this cold period is necessary to slow their metabolism and allows them to survive this period and begin the poleward migration in the spring [12,34]. The monarchs in this experiment were not given the opportunity to migrate to Mexico, and they did not actually reach and stay at the overwintering areas, yet they were still able to recalibrate their magnetic compass based on the temperature cue provided. Increasing temperatures could potentially impact this migration cue; higher overwintering temperatures may inhibit the ability of migratory monarchs to recalibrate their sun compass and magnetic compass and jeopardize their spring remigration to their breeding grounds. Collecting greater knowledge of how orientation cues are established may aid in the preservation of this behavior not only in monarchs, but in other migratory animals [7,35]. Particular attention should be paid to the effects of increasing temperatures at the overwintering sites, as it has a critical role in the reorientation of these mechanisms. Without the coldness cue, monarchs may continue to fly equatorwards without returning north, thereby potentially critically disrupting the annual migratory cycle.

## Materials and methods

### Animals

Adult fall migratory monarch butterflies, *Danaus plexippus* (Linnaeus 1758), of both sexes were collected on-the-wing from September 4^th^ to October 13^th^, 2023 at the University of Cincinnati Center for Field Studies (Harrison, Ohio, USA; 39.28°N, 84.74°W) and at the Cincinnati Nature Center’s Milford Field Station (Milford, Ohio, USA; 39.24°N, 84.19°W). Monarchs were housed under fall-like conditions in a Percival incubator (model: I36LLC8, manufacturer: Percival Scientific, Inc., Perry, Iowa, USA) with a 12h:12h Light:Dark (L:D) cycle (lights on at 06:00; lights off at 18:00), a constant temperature of 21°C during the light phase and 12°C during the dark phase, all at 70% relative humidity [17]. All monarchs were fed a 25% honey solution every other day [36].

### Magnetic orientation trials

The same apparatus and protocol were used as in Kendzel et al. [19]. We examined the orientation behavior of monarchs using a righting apparatus [19] inside of a Helmholtz coil system that allowed us to manipulate magnetic field parameters (inclination angle, total field intensity, polarity) and expose monarchs to artificially generated magnetic fields. Trials were completed in a darkroom, with the righting response apparatus consisting of an open-top black cardboard box (LWH: 30 cm x 33 cm x 36 cm) inside of the coil system, with the top serving as the source of diffused, non-directional light which possessed the necessary parameters that activate magnetosensation in monarchs [18]. A mesh wall (32 cm x 32 cm) was centered in the box, perpendicular to the ground, and served as the plane of movement and provided the ability to orient in all 360 degrees possible for the monarchs during their trials. A plastic clamp was embedded in the wall behind the monarch to hold it via its wings prior to the start of the trial. A camera was placed above the clamps to record all movement throughout the experiment.

We characterized their orientation behavior in three conditions by manipulating the total field intensity and inclination angle: (1) three days flight south of the overwintering site (3DS; Acapulco, Guerrero, Mexico; 16.87°N, 99.88°W), (2) the overwintering site (OWS; center of the Monarch Butterfly Biosphere Reserve, Michoacán-Mexico State, Mexico; 9.56°N, 100.29°W), (3) three days flight north of the overwintering site (3DN; San Luis Potosi, San Luis Potosi, Mexico; 22.16°N, 100.97°W). These locations were selected because of the predicted distance of flight that a monarch would roughly achieve in three days, given that a monarch travels roughly 100 km per day during their fall migratory period [25,37]. This type of trial condition is consistent with how other migratory animals that have a magnetic map are tested (e.g., sea turtles [4] and salmonid fishes [5]). Considering that some migratory animals that possess a magnetic map can course correct in displacement trials [38], if monarchs were utilizing a general magnetic map sense, they would also adjust their course if they were as much as three days off course. All monarchs were first tested at 3DS magnetic conditions and then tested in reverse order (3DS, OWS, and then 3DN) so that they would be completely unfamiliar with conditions, and it would not be a part of their normal migratory route.

Before a trial, a monarch was placed in a mesh cage inside a coil system set to the magnetic condition that they would be tested during a trial, to allow for one hour of free movement and acclimation. In the Helmholtz coil system, the horizontal coil allowed us to produce a field that aligned with the monarch’s axis of rotation and position within the apparatus; magnetic south (mS) was on the right side and magnetic north (mN) was on the left side (note: Figs 1 and 2 show this in reverse to be consistent with Kendzel et al. [19]). Trial magnetic conditions were measured and calibrated using the Physics Toolbox phone application at the head position of the butterfly before each trial. The magnetic parameters of each site were set to Earth-strength (38.22–41.37μT). Magnetosensation trials were completed under diffused light (light transmitted through a diffuser; 250 W 4-in-1Work light, LG Sourcing, Inc., N. Wilkesboro, North Carolina, USA) with sufficient lighting conditions to activate their magnetosensors (total irradiance 7.02 × 10 13 photons s − 1 cm − 2 nm − 1, containing the relevant wavelengths between 380 and 420 nm; Fig 1C, right) [19,20].

The monarch was clamped for one minute in the head down position before being released and allowed free movement on the vertical plane for a maximum of five minutes. The orientation of the monarch was scored based on the position it maintained until the end of the trial. Orientation position was recorded using the iDVR-Pro digital video recording system (model: IDVR-RRO8A HD2, manufacturer: CCTV Camera Pros, Lantana, Florida, USA) and measured with a protractor from the video recording projection. Scores were noted once the monarch maintained a position for at least one minute. Once all trials at that condition were completed for all monarchs, the coils were then set to the next field position and all monarchs would be tested again after at least 24 hours had passed. The same monarchs were tested in all three conditions using a repeated measures design.

### Coldness experiment

After all fall migratory monarchs completed the orientation trials, they were split into two groups and housed under either fall-like (control; n = 12) or overwintering-like conditions (n = 11) for 24-days [17]. Those housed under fall-like conditions were housed in a DigiTherm incubator (model: DT2-MP-47L, manufacturer: Tritech Research, Los Angeles, California, USA) with a 12h:12h light:dark cycle (lights on at 06:00; lights off at 18:00), a constant temperature of 21°C during the light phase and 12°C during the dark phase, all at 70% relative humidity [17]. The group housed under overwintering-like conditions experienced cold conditions in a DigiTherm incubator with the same light and humidity but a constant temperature of 11°C during the light phase and 4°C during the dark phase [17]. All monarchs continued to be fed a 25% honey solution every other day [36].

We examined all monarch behavior again in the 3DN magnetic parameters in order to assess how monarchs magnetically recalibrated after exposure to overwintering-like coldness. Testing at 3DN was done so that it would be a set of conditions they would likely naturally experience after overwintering.

### Ethics statement

We complied with all relevant institutional and local animal welfare laws, guidelines, policies, and regulatory standards during the course of this study. No specific permits were required for this study. No prior approval or waiver of approval, e.g., from an animal research ethics committee, was required for this study. Animals were not subjected to any form of anesthesia or euthanasia, nor were animals sacrificed, during this study.

### Statistical analysis

We assessed the data using the same methods as Guerra *et al.*[18] and Kendzel *et al.*[19]. The Rayleigh’s test and V-test were used to assess directionality. Rayleigh’s test was used to determine if the butterflies displayed a significant mean group orientation for each trial condition. The V-test was similarly used as an extension of the Rayleigh’s test such that it tested whether or not the significant mean orientation of the group was consistent with the *a priori* expected orientation prior to trials. For multisample tests, we used the Moore-Rayleigh test [39] and Hotelling’s paired test to see if the mean orientations differed across testing conditions. Summary statistics were calculated using the R package ‘circular’ (https://cran.r-project.org/package=circular) and comparisons between trials were calculated using the program Oriana 4 (https://www.kovcomp.co.uk/oriana/; Kovach Computing Services, Pentraeth, Isle of Anglesey, UK). The data used in this study are publicly available and can be found at the University of Cincinnati’s Scholar@UC digital repository (https://doi.org/10.7945/1qbt-dm02). These data at the University of Cincinnati’s Scholar@UC digital repository are made available under the Open Data Commons Attribution License: http://opendatacommons.org/licenses/by/1.0/.
